# Impact of Pharmacist–Psychiatrist Collaborative Patient Education on Medication Adherence and Quality of Life (QOL) of Bipolar Affective Disorder (BPAD) Patients

**DOI:** 10.3389/fphar.2017.00722

**Published:** 2017-10-10

**Authors:** Ambed Mishra, Gudi S. Krishna, Sravani Alla, Tony D. Kurian, Justin Kurian, Madhan Ramesh, M. Kishor

**Affiliations:** ^1^Department of Pharmacy Practice, JSS College of Pharmacy, Jagadguru Sri Shivarathreeshwara University, Mysore, India; ^2^Department of Psychiatry, JSS Hospital, Mysore, India

**Keywords:** BPAD, collaborative care, pharmacist, patient education, medication adherence, quality of life

## Abstract

**Background:** Bipolar Affective Disorder (BPAD) is one of the leading causes of disability globally. Medication non-adherence and low quality of life (QOL) are the major challenges associated with the treatment of BPAD patients.

**Objective:** Aim of this study was to assess the impact of pharmacist–psychiatrist collaborative patient education on medication adherence and QOL of BPAD patients.

**Methodology:** A prospective randomized control study was conducted in the psychiatry outpatient department in a tertiary care setting. The eligible patients were enrolled and randomized into test (collaborative care) and control (usual care) groups. Patient education was provided by pharmacists to the test group patients, along with the usual care provided to all the patients. Patients were followed for three follow-ups of nearly 1 month intervals. Medication adherence and QOL were assessed by Medication Adherence Rating Scale and WHOQOL-BREF questionnaire, respectively. *T*-test was used and *P*-values < 0.05 were considered statistically significant.

**Results:** Out of 75 patients enrolled, 73 patients were followed for all the three follow-ups and completed the study. Thirty-eight patients belonged to test and 35 were in control group. The mean age of patients was 34.21 ± 10.91 years. Forty-eight (65.75%) patients belonged to age group of 18–39 years. There were 41 males (56.16%) and 32 female patients (43.83%) in the study. Mean improvement in medication adherence and QOL of the test and control groups were found to be 2.06 ± 0.15 (<0.001) and 13.8 ± 10.5 (<0.05), respectively.

**Conclusion:** This study concluded that pharmacist–psychiatrist collaborative patient education can significantly improve the medication adherence and QOL of the BPAD patients. Statistically significant results indicating improved patient care and outcomes were possible when pharmacists worked as a team with psychiatrists.

## Introduction

Bipolar Disorder or Bipolar Affective Disorder (BPAD) causes unusual changes in mood, energy, activity levels and the ability to carry out routine tasks (American Psychiatric Association, 2013). It is also called as manic-depressive illness (Russell and Browne, 2005; American Psychiatric Association, 2013). Both manic and depressive episodes are separated by periods of normal mood and manic episodes which usually involve elevated or irritable mood, over-activity, pressure of speech along with inflated self-esteem and decreased need for sleep. The patients who have manic attacks but do not experience depressive episodes are also diagnosed as BPAD (Medscape, 2017). BPAD affects about 5.7 million American adults, or about 2.6% of the United States population every year who are 18 and older (World Health Organization, 2001; American Psychiatric Association, 2013). BPAD is a leading cause among all the factors responsible for disability globally (World Health Organization, 2001).

Medication non-adherence and decreased quality of life (QOL) are major concerns in BPAD patients. Patient education provided by pharmacists is an important source of medication information for patients diagnosed with various psychiatric disorders and their carers as well (Ellicott et al., 1990). Pharmacists play a vital role in providing care to psychiatric patients related to medication therapy management and providing patient education during the therapy as well (Ormel et al., 2008; Dobscha et al., 2016). Collaborative care improves patient’s health significantly when pharmacists work in collaboration with physicians to manage patient care (American Society of Health-System Pharmacists [ASHP], 1997; Phokeo et al., 2004; Chisholm-Burns et al., 2010).

Pharmacists can play a major role in patient care but access to patient care services which can be delivered by pharmacists is limited by policy and compensation barriers (Giberson et al., 2011). Strategies for expanding pharmacists’ patient care services through team-based collaborative practice agreement (CPA) were proposed by a study group in United States which would enable pharmacists to strengthen partnerships with different healthcare providers to improve patient care in psychiatric department (Nichols, 2002). Pharmacists have the potential to ensure optimal pharmacotherapeutic outcomes for their patients in addition to the patient counseling and ensuring prompt access to good quality medications (Akvardar et al., 2006). Psychiatric pharmacists play an essential role in counseling patients and ensuring that the proper treatment outcomes are achieved in terms of patient’s medication adherence which results in improved QOL (Enato and Aina, 2010). In United States, collaborative care including pharmacists are common, where the American College of Physicians with the American Society of Internal Medicine had jointly agreed on the scope of practice for pharmacists which supports an expanded role which includes pharmacist-physician collaborative agreements (Chisholm-Burns et al., 2010). Pharmacist–Psychiatrist collaborative care is a new concept in most of the developing countries including India (American Society of Health-System Pharmacists [ASHP], 1997; Chisholm-Burns et al., 2010). In India, providing medication usage information during the dispensing of medication to individuals with mental disease is minimal and there are only a few clinical pharmacists who could provide patient counseling to help prevent the medication non-adherence, treatment discontinuation and related complications, which is commonly observed in psychiatric patients (Ormel et al., 2008; Dobscha et al., 2016).

The purpose of our study was to compare the impact of pharmacist–psychiatrist collaborative patient education with that of treatment by psychiatrist alone for BPAD patients.

## Materials and Methods

A prospective randomized controlled study was conducted in the outpatient department of Psychiatry in a tertiary care setting in South India over a period of 6 months. The approval was obtained from the local Institutional Human Ethical Committee of JSS University, JSS College of Pharmacy, Mysore for conducting the study. All subjects gave written informed consent in accordance with the Declaration of Helsinki prior to enrollment in this study.

### Study Procedure

The patients who visited the psychiatry outpatient department of either sex, aged ≥18 years, treated for BPAD and literate, were included in our study. The patients diagnosed with BPAD with other co-morbidities were excluded from this study. All the relevant data for the evaluation were collected from out-patient case records, patient prescriptions, communicating with healthcare professionals and by interviewing the patient and carers. BPAD patients who met the study criteria were included into the study in consultation with psychiatrist after obtaining the informed consent from the patients. The subjects who were enrolled into the study were grouped into collaborative care (test) and usual care (control) groups by using simple randomization technique. Patient education was provided only to the test group by pharmacists along with the usual care given to all the patients in both groups.

Treatment options for management of BPAD can be broadly classified as mood stabilizers, antidepressants, antipsychotic medications, electroconvulsive therapy (ECT), adjunctive medications and psychosocial interventions. Use of various treatment options is guided by the phase of illness including mania, hypomania, depression or mixed, in which patient presents to the clinician (Avasthi et al., 2004; Shah et al., 2017). Clinical pharmacy related services including patient education results in better outcomes from the therapy including improvement of medication adherence and QOL of patients (Parthasarathi et al., 2004).

Usual care by psychiatrists involved an examination of patient’s disease and prescribing medications during the consultation session. Patient education provided by the pharmacist in this study included patient and carers awareness of the medications prescribed, disease, importance of adherence to medications and impact on overall QOL. Leaflets were used during patient education. Patient education complemented with suitably designed patient information leaflets (PILs) has greater impact on the knowledge, attitude and practice of the patients toward their disease management (Adepu and Swamy, 2012).

Patient-specific education with PILs was provided to each patient for better impact on medication adherence behavior and QOL. All the enrolled BPAD patients or their carers were contacted by the research pharmacist at least a week before their follow-up date and also a day before the follow-up by telephone to remind them of the follow-up date at the hospital. This helped in ensuring that enrolled patients do not miss their follow-up. Patients were followed for three follow-ups and during each follow-up, the medication adherence and QOL were assessed by using Medication Adherence Rating Scale (MARS) and World Health Organization Quality of Life (WHOQOL-BREF) questionnaire, respectively. The test group patients were provided with patient education session during each follow-up to improve their medication adherence and QOL. Patient feedback about the reason for their non-adherence was obtained during each follow-up. If a patient in test group was found non-adherent, patient education session that included importance of adherence to medication in BPAD along with motivational support was provided. Carers were also provided with counseling when patients were found to be non-adherent and were educated about the importance of medication adherence on the patient’s condition. The obtained results were compared between test and control groups for each follow-up.

### Statistical Analysis

The collected data for BPAD patients was analyzed using SPSS version 20 for statistical analysis. Descriptive statistics on sample characteristics were computed, including means, standard deviation, and frequency distributions. The differences between means were calculated using individual *T*-test. *P*-values less than 0.05 were considered statistically significant.

### Development of Patient Information Leaflets (PILs)

Patient information leaflets specific to BPAD were developed by the investigator pharmacists in consultation with the psychiatrists to educate the patients with relevant disease conditions. The prepared PILs were reviewed by the team of four psychiatrists and two clinical pharmacists. After the review process, based on the feedback by the reviewers, the PILs were further modified as appropriate and finalized. The approved PILs were translated into the local language (Kannada) by linguistic experts. The PILs were used in the patient and carers education as an additional material for the counseling session.

## Results

### Patient Demographics

A total of 89 patients diagnosed with BPAD met the study criteria, 75 patients agreed to enroll into the study and 73 patients completed the study. Initially, 38 patients were assigned to test and 37 patients were assigned to the control group. Two patients from the control group did not turn-up for the next follow-up after enrollment and were considered as drop-outs. Of the total 73 patients who completed the study, 38 were from test and 35 from control group. Data collected from only those patients who completed the study for all the follow-ups was analyzed. Majority of the study subjects belonged to the age group of 18–39 years (*n* = 48; 65.75%) and the least in the age group of ≥60 years (*n* = 2; 2.73%). The mean age of the study patients was found to be 34.71 ± 10.65 and 33.71 ± 11.17, for the test group and control group, respectively. Also, majority of the patients were male (*n* = 41; 56.16%) followed by females (*n* = 32; 43.83%) in our study. Demographic details of the study patients are presented in **Table 1**.

**Table 1:** Demographic details of study population.

Gender	Age group (years)								
	18–39			40–59			≥60		
	Control	Test	Total	Control	Test	Total	Control	Test	Total
Male (*n*)	13	13	26	7	6	13	1	1	2
Female (*n*)	11	11	22	3	7	10	0	0	0
**Total**	24	24	48	10	13	23	1	1	2

### Medication Adherence

The medication adherence assessment is presented in **Table 2**.

**Table 2:** Comparison of medication adherence scores.

Follow-up	Control (Mean ±*SD*)	Test (Mean ±*SD*)	*P*-value^∗^
First follow-up	4.66 ± 0.83	5.44 ± 0.55	<0.001
Second follow-up	5.05 ± 0.72	6.42 ± 0.59	<0.001
Third follow-up	5.25 ± 0.51	7.31 ± 0.66	<0.001

**^∗^***T-test*.

## Control Group

Assessment of the patient’s medication adherence from 1st follow-up to 2nd follow-up and 2nd follow-up to 3rd follow-up showed a mean improvement in medication adherence level of 0.39 ± 0.11 and 0.2 ± 0.21, respectively. The mean improvement in medication adherence (1st follow-up to 3rd follow-up) in the control group was found to be 0.59 ± 0.32.

## Test Group

Similarly, assessment of patient’s medication adherence from 1st follow-up to 2nd follow-up and 2nd follow-up to 3rd follow-up showed a mean improvement in medication adherence level of 0.98 ± 0.04 and 0.89 ± 0.07, respectively. The mean improvement in medication adherence (1st follow-up to 3rd follow-up) in the test group was found to be 1.87 ± 0.11.

## Between Control and Test Groups

The improvement in medication adherence observed in this study was 2.06 ± 0.15 in the test group, indicating improved medication adherence outcomes when pharmacists provided patient education in addition to the usual care by psychiatrists.

## Quality of Life (QOL)

The comparison of QOL scores has been presented in **Table 3**.

**Table 3:** Comparison of QOL scores.

Item	Follow-up	Domains	Control (Mean ±*SD*)	Test (Mean ±*SD*)	*P*-value^∗^ (<0.05)
WHO-BREF SCALE	**First follow-up**	Domain 1	32.54 ± 9.75	33.94 ± 11.38	**0.575 NS**
(Quality of life)		Domain 2	27.34 ± 12.57	34.21 ± 14.46	**0.034**
		Domain 3	34.6 ± 7.71	38.73 ± 12.33	**0.093 NS**
		Domain 4	35.65 ± 6.16	40 ± 11.32	**0.048**
		**Overall score**	**32.53 ± 9.04**	**36.72 ± 12.37**	**-**
	**Second follow-up**	Domain 1	44.14 ± 8.69	52.07 ± 10.38	**0.001**
		Domain 2	40.65 ± 8.57	53.21 ± 10.33	**0.001**
		Domain 3	43.8 ± 9.91	49.5 ± 15.17	**0.064NS**
		Domain 4	43.94 ± 9.36	50.94 ± 13.13	**0.011**
		**Overall score**	**43.13 ± 9.13**	**51.43 ± 12.25**	**-**
	**Third follow-up**	Domain 1	45.08 ± 9.25	56.71 ± 16.72	**0.001**
		Domain 2	41 ± 8.88	60.23 ± 16.59	**0.001**
		Domain 3	42.68 ± 11	54.1 ± 26.14	**0.019**
		Domain 4	45.2 ± 9.32	58.15 ± 20.98	**0.001**
		**Overall score**	**43.49 ± 9.61**	**57.29 ± 20.11**	**-**

^∗^*T-test; NS, not significant. Domain 1: Physical health; Domain 2: Psychological health; Domain 3: Social relationships; Domain 4; Environmental quality of life*.

### Control Group

Assessment of patient’s quality of life from 1st follow-up to 2nd follow-up and 2nd follow-up to 3rd follow-up and 1st follow-up to 3rd follow-up showed a mean improvement in 10.6 ± 0.09 and 0.36 ± 0.48 and 10.96 ± 0.57, respectively.

### Test Group

Assessment of patient’s quality of life from 1st follow-up to 2nd follow-up and 2nd follow-up to 3rd follow-up and 1st follow-up to 3rd follow-up showed a mean improvement in 14.71 ± 0.12 and 5.86 ± 7.86 and 20.57 ± 7.74, respectively.

### Between Control and Test Groups

Assessment of QOL between both groups showed mean improvement of 13.8 ± 10.5 in the test group. It showed better outcomes for QOL when pharmacists provided patient education in addition to the usual care by psychiatrists.

## Discussion

Improvement in medication adherence and QOL were observed in all the follow-ups in the test group when compared the scores of both groups by using Medication Adherence Rating Scale (MARS). Overall, there was statistically significant improvement in medication adherence of test group patients in individual follow-ups and overall mean medication adherence as well, when compared to the control group where only the overall improvement in mean score for medication adherence was statistically significant. This clearly showed that the collaborative care approach was better in increasing the medication adherence over the usual care approach which has been traditionally been used. Similar finding was observed in a study carried out by Offor (2011) wherein the educational intervention provided by the pharmacists resulted in an improvement in patients’ mean medication adherence.

Patient feedback about the possible reasons for their non-adherence was obtained during each follow-up. Based upon the feedback, it was observed that forgetfulness, adverse effects and medication cost were the major reasons associated with the medication non-adherence in majority of the patients in the study. In few of the cases, lack of information on disease and medications, lack of disease concern and social support were the reasons for the medication non-adherence. This result is similar to the study by Nirojini et al. (2014) wherein the reasons for non-adherence included adverse effects and medication cost followed by increased number of medications, unavailability and forgotten by the patient representatives.

There was significant improvement in the mean medication adherence and QOL scores of the test group when compared to that of the control group (**Tables 2**, **3**). This clearly showed improvement in the medication adherence and QOL of the BPAD patients in test group which meant that the pharmacist–psychiatrist collaborative patient education is effective in treatment of BPAD patients. Similar findings were reported by Galuppi et al. (2010) wherein positive outcomes in QOL of patients was observed in the study.

To the best of our knowledge, this study appears to be the first of its kind in India, addressing an almost neglected and often completely unmentioned area of pharmacy practice in the country. We believe that BPAD patients should have the opportunity of being evaluated for the quality of medical and pharmaceutical care that they receive. Moreover, it is the responsibility of the pharmacist to provide the counseling to patients with BPAD and their carers as well (if required) on appropriate use of medications to improve the medication adherence rate and aid to improve their quality of life as well.

## Author Contributions

AM, GK, SA, TK, JK, and MR provided patient education, MK provided usual care to all the patients, and all the authors contributed to the acquisition of the data. AM, MR, and MK contributed to the analysis of the data, made substantial contributions to the conception or design, and interpreted data for the work. AM drafted the work. AM, and MK critically revised the work, for important intellectual content. All authors listed have made a substantial, and direct contribution to the work, and approved it for publication.

## Conflict of Interest Statement

The authors declare that the research was conducted in the absence of any commercial or financial relationships that could be construed as a potential conflict of interest.
